# Molecular docking analysis of α-Topoisomerase II with δ-Carboline derivatives as potential anticancer agents

**DOI:** 10.6026/97320630017249

**Published:** 2021-01-31

**Authors:** Selvaraj Ayyamperumal, Dhananjay DJ, Vyshnavi Tallapaneni, Surender Mohan, Basappa S, Jubie Selvaraj, Nanjan Moola Joghee, Chandrasekar MJN

**Affiliations:** 1 Department of Pharmaceutical Chemistry, JSS College of Pharmacy, JSS Academy of Higher Education & Research, Ooty, Nilgiris - 643001,Tamil Nadu, India; 2 PG Studies and Research, JSS College of Pharmacy, JSS Academy of Higher Education & Research, Ooty, Nilgiris - 643001, Tamil Nadu, India; 3 Laboratory of Molecular Biology and Genetic Engineering, School of Biotechnology, Jawaharlal Nehru University, New Delhi - 110067, India; 4 Department of Studies in Organic Chemistry, University of Mysore, Manasagangotri, Mysore - 570006, Karnataka, India

**Keywords:** Drug design, ADMET, Molecular Docking, Molecular Dynamics, α-Topoisomerase II, Anticancer agents

## Abstract

The enzyme, α-topoisomerase II (α-Topo II), is known to regulate efficiently the topology of DNA. It is highly expressed in rapidly proliferating cells and plays an important role in replication, transcription and chromosome organisation. This has prompted several investigators to pursue α-Topo II inhibitors as anticancer agents. δ-Carboline, a natural product, and its synthetic derivatives are known to exert potent anticancer activity by selectively targeting α-Topo II. Therefore, it is of interest to design carboline derivatives fused with pyrrolidine-2,5-dione in this context. δ-Carbolines fused with pyrrolidine-2,5-dione are of interest because the succinimide part of fused heteroaromatic molecule can interact with the ATP binding pocket via the hydrogen bond network with selectivity towards α-Topo II. The 300 derivatives designed were subjected to the Lipinski rule of 5, ADMET and toxicity prediction. The designed compounds were further analysed using molecular docking analysis on the active sites of the α-Topo II crystal structure (PDB ID:1ZXM). Molecular dynamic simulations were also performed to compare the binding mode and stability of the protein-ligand complexes. Compounds with ID numbers AS89, AS104, AS119, AS209, AS239, AS269, and AS299 show good binding activity compared to the co-crystal ligand. Molecular Dynamics simulation studies show that the ligand binding to α-Topo II in the ATP domain is stableand the protein-ligand conformation remains unchanged. Binding free energy calculations suggest that seven molecules designed are potential inhibitors for α-Topo II for further consideration as anticancer agents.

## Background

Topoisomerases play an important role in regulating cellular processes such as replication, transcription and chromosomal segregation by altering DNA topology [1-4]. Type I topoisomerases (Topo I) modify the DNA topology in an ATP dependent fashion by creating single strand breaks in DNA whereas type II topoisomerases (Topo II) do so by creating double strand breaks in DNA [5]. Topo II is a well-known anticancer target and some of the most effective anticancer agents currently used target Topo II [6]. Topo II chemotherapy (treating with etoposide, doxorubicin and their analogues), however, is associated with toxic side effects and secondary malignancies [7]. These drugs, however, show potent anticancer activity without any secondary malignancies when the sub type of TopoII, namely α-Topo II is targeted [4]. The expression of α-Topo II is believed to be tightly linked to the actively replicating cancer cells and its level changes during the cell cycle [8,9]. It has, therefore, been suggested that designing more specific drugs targeting only α-Topo II without stimulating β-Topo II which cause chromosome rearrangements, may be beneficial for cancer treatment [10,11]. α-Topo II concentration is known to increase 2-3 fold during G2/M phase of the cell cycle and orders of magnitude are higher in rapidly proliferating cells than in quiescent cell populations [12,13]. After binding to DNA, it produces a double-strand DNA break by nucleophilic attack on a pair of tyrosine residues [14,15]. α-Topo II assumes two different conformations, resembling an open clamp in the absence of ATP and a closed clamp in the presence of ATP. The open conformation binds two segments of DNA, forming the pre-cleavage complex. These segments are nicked by the enzyme (G segment) and transported (T segment) to unwind the supercoiled DNA [16]. Agents that target α-Topo II are, therefore, efficacious, and safe anticancer drugs with reduced risk of secondary malignancies. The anthracyclines are amongst the most widely used α-Topo II inhibitors and this proven ability of α-Topo II to efficiently regulate the topology of DNA has, therefore, prompted many research groups to pursue inhibitors of α-Topo II for cancer research. δ-Carbolines are heterocyclic compounds with a broad spectrum of biological activity including antimuscarinic [17], antihyperglycemic [18], antimalarial, antiplasmodial [19], antifungal, anticryptococcal, antiviral [20] and anticancer activity [21]. Though δ-Carbolines containing several other scaffolds have been designed, synthesized and evaluated, to the best of our knowledge, δ-Carboline derivatives fused with pyrrolidine 2,5-dione (succinimide) have not been reported so far, possibly due to the lack of expedient synthetic methods. Pyrrolidine 2,5-diones fused with δ-Carbolines are of interest because the succinimide part of the fused polycyclic hetero aromatic molecules can interact with the ATP binding pocket via the hydrogen bond network with selectivity towards α-Topo II. We report insilico design of some novel δ-Carboline derivatives fused with pyrrolidine 2, 5dione with synthetic accessibility and capable of binding to α-Topo II. These molecules were investigated for their ADMET properties, hit identification, molecular docking, molecular dynamics, and free energy binding. Among the 300 molecules designed, seven molecules were identified as potential inhibitors of α-Topo II.

## Materials and Methods:

### Designing of compounds

**Figure 1:**
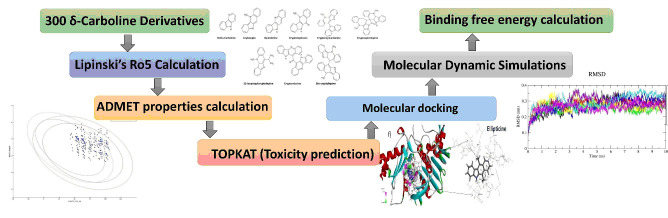
Overview of the study design.

Ligand-based drug design is an indirect approach to facilitate the development of pharmacologically active compounds by studying molecules that interact with biological targets of interest [22]. In the present study, our designing process for anticancer agents started with the selection of suitable δ-carboline scaffold to which recognition of elements (methyl, ethyl, benzyl, benzoyl, pyridine, 1,3,4-triazole, acetic acid, propionic acid, 2-methylbutanoic acid, 4-methylpentanoic acid, 4,6-dimethylpyrimidine and benzoic acid) are substituted that are predicted to interact with α-Topo II [23,9,24-26]. We designed molecules based on synthetic accessibility and possible combinations of scaffold and substituents, to provide a good fit, and hence a proper screening hit. Derivatives of two δ-carboline scaffolds possessing pyrrolidine-2,5-dione at different positions were designed (Figure 3). The structure of δ-carbolines fused with pyrrolidine-2,5-dione was drawn using ACD/ChemSketch Freeware (www.acdlabs.com) and saved in MDL-mol. The file was then introduced into Discovery Studio (DS 4.1) in structure data format (SDF) for further in silico studies. The overall protocol of the study is given in Figure 1.

### Preparing ligands:

The standard formal charges on functional groups are essential in the design of molecules. Preparing ligands is directing the designed ligands to tautomerizing the amide groups and indicating the ionization state for compounds physiological pH (pH=7.4) in the calculation at Kekule form. The 3D-structure of δ-carbolines were cleaned and prepared for ADMET analysis, Molecular docking (Libdock, Cdocker) using the protocol "Prepare Ligands" in DS 4.1 [27].

### Lipinski's rule of five parameters:

Compound flexibility, molecular size, and hydrophobicity are known to have a profound effect on living organisms. The physicochemical property of a drug such as absorption depends simultaneously on the dose, solubility and permeability. Failure to take these into consideration, influenced the high attrition rates observed in the first combinatorial libraries but later contributed to Lipinski's Ro5 guidelines in drug discovery. Ro5 has perhaps been the most crucial concept in preclinical drug discovery during the last two decades [28,29]. Discovery Studio 4.1 was used to assess the molecular parameters of the designed compounds.

### ADMET studies:

The computational ADMET prediction (absorption, distribution, metabolism, excretion, toxicity) and TOPKAT (Toxicity prediction by computer-assisted technology) are constitutive methods used in modern drug discovery to predict the drug pharmacokinetics and toxicity. These studies predict ADMET properties of the designed molecules and help in the structural refinements to improve ADME and remove toxicities. ADMET properties are necessary for the selection and development of drug candidates. ADMET properties for the designed δ-Carboline derivatives were estimated using Discovery Studio 4.1. The properties of human intestinal absorption (HIA) after oral administration, aqueous solubility, blood-brain barrier (BBB) penetration after oral administration, CYP2D6 enzyme inhibition using 2D chemical structure, potential organ toxicity for the structurally diverse compounds designed and whether a compound is likely to be highly bound (>= 90% bound) to the carrier protein in the blood, were predicted for all the screened structures. Toxicity was predicted in male, female mouse and rat to calculate carcinogenity, Weight of Evidence, AMES, Developmental Toxicity Potential, Rat Oral Dose, Mouse Carcinogenic Potency, Rat Carcinogenic Potency, Rat maximum tolerated dose, Rat inhalation, LOAEL (Lowest observed adverse effect level), Fat head minnow, Daphnia, Biodegradability, Skin Irritancy, Skin sensitization and Ocular skin irritancy.

### Protein preparation:

**Figure 2:**
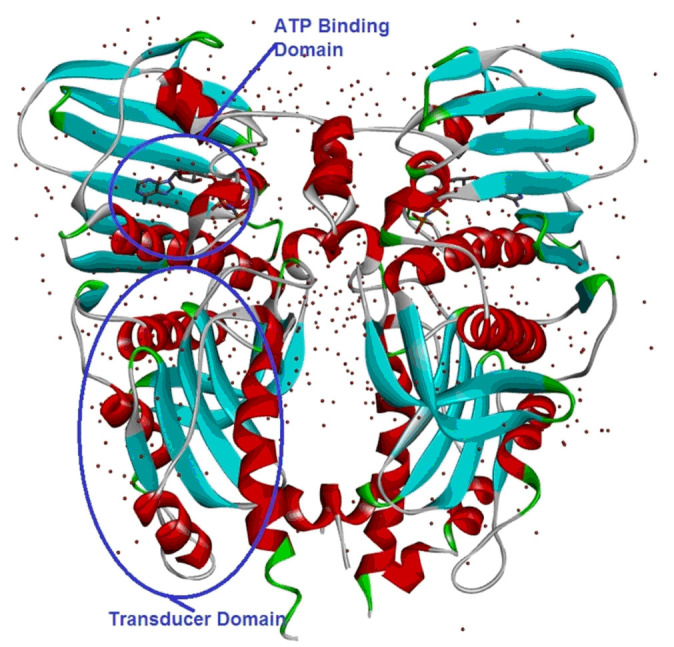
The 3D-Dimensional structure of α-Topo II (PDB.ID.1ZXM).

**Figure 3:**
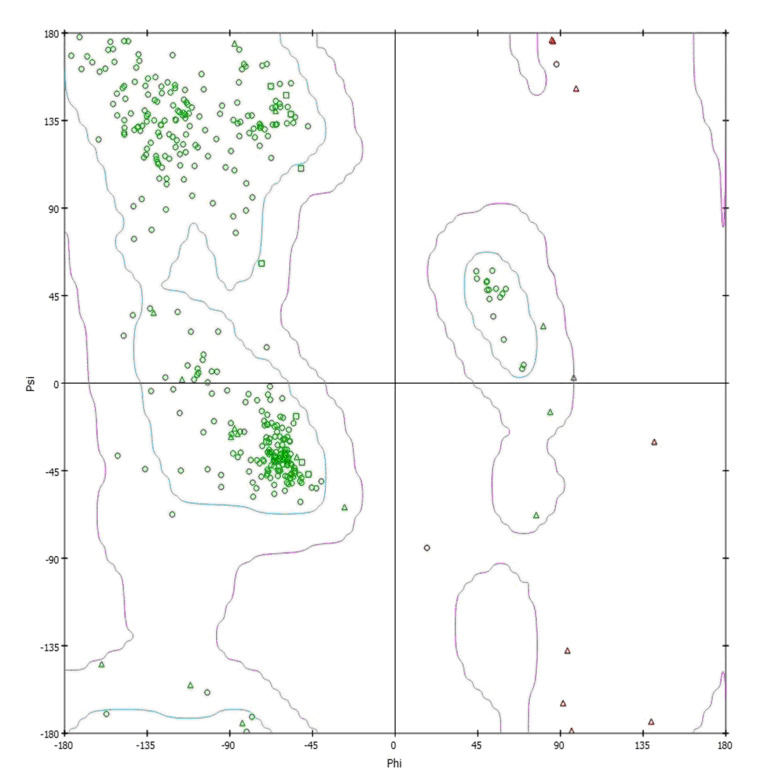
Ramachandran plot of the prepared protein structure (PDB ID: 1ZXM).

The structure of α-Topo II (PDB ID: 1ZXM) was retrieved from the Protein Data Bank (http://www.pdb.org) Figure 2. The protein preparation was performed using Discovery Studio 4.1 program by the missing atoms in incomplete residues, modelling missing loop regions, deleting alternate conformations (disorder), removing water molecules, standardizing atom names, and protonating titratable residues by using the predicted pKa. The prepared protein was validated using Ramachandran plot analysis (Figure 3).

### Binding site identification:

In α-Topo II protein, the N-terminal domain contains the ATPase domain (about 1-265residues), the transducer domain (about 266-428 residues) and the toprim domain (455-572 residues). The ATP binding domain is responsible for the anticancer activity through the binding of organic cyclic compounds [30].

### Virtual docking, grid-based docking and flexible docking:

Libdock robust and rigid molecular docking was performed to identify hit molecules using Accelrys Discovery Studio 4.1. Libdock identifies the hits as lead identification using rapid docking of chemical libraries of compounds [31]. The advantage of this method is to retrieve the active compound from the diverse compound collection. Cdocker program and Autodockvina are used for molecular docking for the identified hit molecules from libdock. Docking enables us to understand the molecular interactions, those that take place between the ligand and the corresponding receptor. AutoDock Tools (ADT) 1.5.4 was used to prepare all the input files. Kollman charges method was used for adding Polar hydrogens and partial atomic charges. The α-Topo II structure was saved in PDBQT format to be delivered to AutoDock tools as an input file. The number of a grid point in xyz 98x96x94 (x, y, and z) and grid box center is 35.354x2.159x19.653 (x, y, and z) were then assigned to the α-Topo II binding pocket with the spacing of 0.375Å. All docking calculation parameters were kept as a default value. Ligands were docked using the Lamarckian Genetic Algorithm with initial population of 150 randomly placed individuals, a maximum number of 2500000 energy evaluations, a mutation rate of 0.02 and a crossover rate of 0.8. A total 10 docking confirmations were generated for each selected compound. The grid maps were calculated using Autogrid4 and docking procedure was performed using Autodock4. The structures of the lowest binding energy conformation of the compounds were selected to find the molecular interactions between the receptor and ligands using PLIP.

### Molecular dynamic simulations:

Molecular dynamics (MD) simulation provides detailed information concerning the dynamics of the performance of atoms and molecules. In the present study, MD simulations were performed using GROMACS MD 4.6.5 for the protein-ligand complex through gromos and 54a7 force-field generated protein topology. The initial orientation of the ligand-protein was obtained from previous flexible docking studies for MD simulations. All the systems were solvated using a simple point charge model in the cubic box, and the PRODRUG online tool created topologies for the carboline derivatives. The protein-ligand complex was put in a triclinic box, and the complex structure was solved with simple point charge (spc216), water. Cl- ions were then added to neutralize the system. The system was then relaxed through the energy minimization process. Electrostatic interactions were estimated by using the PME algorithm. Temperature and pressure were stabilized with NVT and NPT. MD simulation was used to generate the final protein-ligand structures after 30ns of simulation time. MD simulations with reasonable initial velocity follow the path of steepest descent on the potential energy surface to a local minimum. The root mean square deviation (RMSD), root mean square fluctuation (RMSF), and the Radius of gyration (Rg) were calculated by g_rms, g_rmsf, and g_gyrate, respectively.

### Binding free energy analysis:

Free energy calculation analysis is useful in drug discovery process as it provides a quantitative estimation of the binding free energies. Binding free energies were calculated for the selected compounds using the g_mmpbsa tool. The molecular mechanics energies combined with Poisson-Boltzmann or generalized Born and surface area continuum solvation (MM/PBSA and MM/GBSA) method was used to estimate the free energy of binding of the ligands to the protein (complex). The binding free energy of the protein-ligand complex was calculated using the MM-PBSA method. 30ns MD trajectory was used for the calculation of MM-PBSA. The binding energy calculations were performed for 500 snap shots taken at an interval of 1000 ps during the stable 30 ns period of MD trajectory.

### Data analysis:

The ligand-protein interaction was analyzed and visualized through Discovery studio 4.1, AutoDock ADT, and PyMOL. Docking pose and MD simulation figures were generated using PyMOL. RMSD, RMSF and the Gyration graph generated through xmgrace.

## Results and Discussion:

### Designing of compounds:

**Figure 4:**
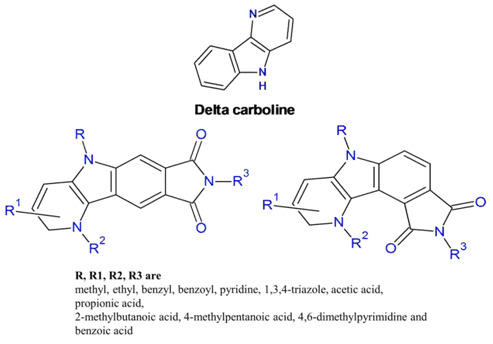
δ-carboline derivatives containing pyrrolidine-2,5-dione.

A novel strategy for the synthesis of δ-carbolines containing pyrrolidine-2,5-dione has been developed in our laboratory. Based on this strategy, 300 new δ-carboline derivatives were designed by modifying the two scaffolds (Figure 4) that possess δ-carbolines fused with pyrrolidine-2,5-dione at different positions. A Sci-Finder database search was carried out for all the designed molecules. All the molecules were found to be novel molecules.

### Lipinski's rule of five parameters:

**Figure 5:**
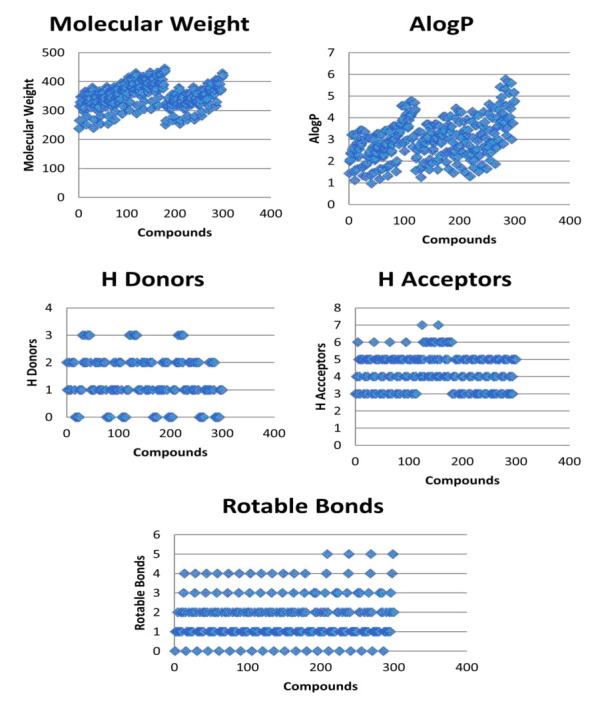
Molecular properties of designed δ-carboline derivatives.

The molecular properties of the designed 300 compounds, AS01 to AS300, were calculated. The molecular weight ranged between 237 (AS01) to 445 (AS179). The Alog P values ranged from 0.958 to 5.767. The number of hydrogen bond donors ranged from 0 to 3. The number of hydrogen bond acceptors ranged from 3 to 7. The rotatable bond ranged from 0 to 5. The designed δ-carbolines derivatives, therefore, pass Lipinski's rule of five. The results are shown in charts (Figure 5).

### ADME and toxicity studies:

**Figure 6:**
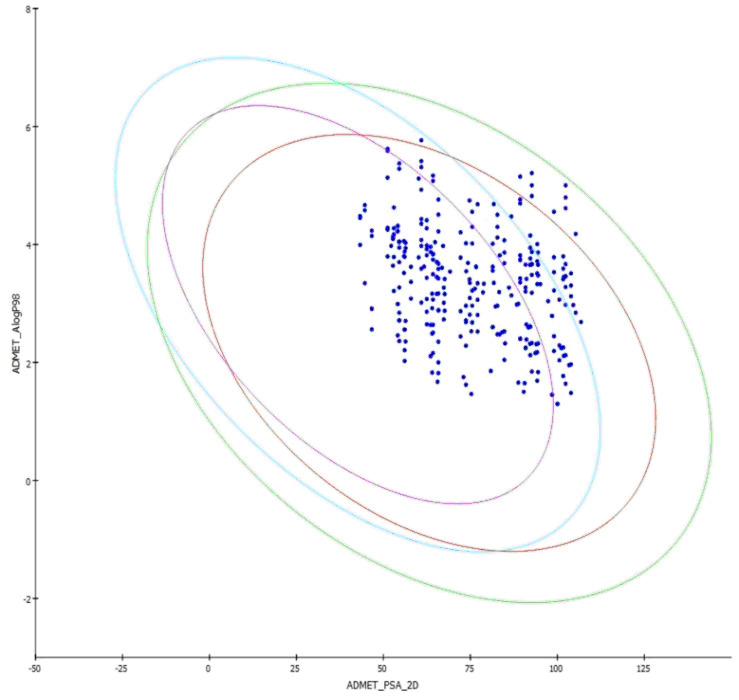
ADMET plot of the designed δ-carboline derivatives.

The results of the ADME and Toxicity studies performed on the designed compounds are given in Supplementary Tables 1 and 2 (see PDF). Compounds AS01, AS11, AS26, AS31, AS41, AS46, AS56, AS61, AS71, AS86, AS104, AS119, AS131, AS146, AS161, AS211, AS221, and AS236 were soluble in water at 25°C. Compounds AS111, AS274, AS284, AS289, and AS294 showed extremely low solubility. The absorption in 95% ellipse and 99% ellipse are shown in ADMET Plot (Figure 6). All the compounds were absorbed moderate to well in human intestinal absorption (HIA). The HIA and blood-brain barrier (BBB), obtained with ADMET_PSA_2D, range from 43.302 in the compound AS259 to 106.825 in the compound AS35.ADMET_AlogP98 ranges from 1.296 in the compound AS219 to 5.767 in the compound AS284. Compounds, AS289 and AS294, are highly penetrant to BBB. The BBB of compounds AS05, AS13, AS14, AS15, AS35, AS43, AS44, AS45, AS74, AS95, AS97, AS100, AS105, AS118, AS120, AS133, AS148, AS164 and AS281 are undefined. The cytochrome P450 mono-oxygenase (CYP) enzymes play a crucial role in drug metabolism. The inhibition or induction of P450 enzyme is one of the most vital causes of metabolic drug interaction and none of the compounds inhibit CYP2D6. Most of the compounds were hepatotoxic to liver except AS89, AS104, AS119, AS134, AS159, AS164, AS174, AS179, AS209, AS234, AS239, AS264, AS269 and AS299. Plasma Protein Binding (PPB) has a vital role in drug distribution. Compounds AS01, AS02, AS03, AS05, AS07, AS10, AS11, AS12, AS16, AS18, AS181, AS196, AS22, AS25, AS26, AS27 and AS31 were found to be non-binding to PPB and the rest of the compounds were binding to PPB. Toxicity (TOPKAT) studies suggest that most of the compounds are non-carcinogenic to Mouse NTP Model (Male/Female) except compounds AS03, AS33, AS38, AS48, AS63, AS76, AS123, AS127, AS128, AS136, AS138, AS151, AS153, AS183, AS198, AS212, AS213, AS223, AS224, AS228, AS231, AS241, AS243, AS258, AS273 and AS288. Most of the compounds are also non-carcinogenic in Rat NTP male/ female, Mouse FDA male/female and Rat FDA male/female models. The Weight of Evidence (WoE) of most of the compounds is non-carcinogen except compounds, AS94, AS97, AS142, AS151, AS153, AS154, AS155, AS156, AS157, AS159, AS163, AS164, AS166, AS168, AS169, AS172, AS247, AS248, AS292 and AS293. The compound, AS99, showed the lowest TD50 value with 2.42 mg/kg/day in mouse. The compound AS01 showed the highest LC50 value with 0.18g/l. The compound AS290 had the predicted EC50 value of 0.63mg/l.

### Molecular docking:

**Figure 7:**
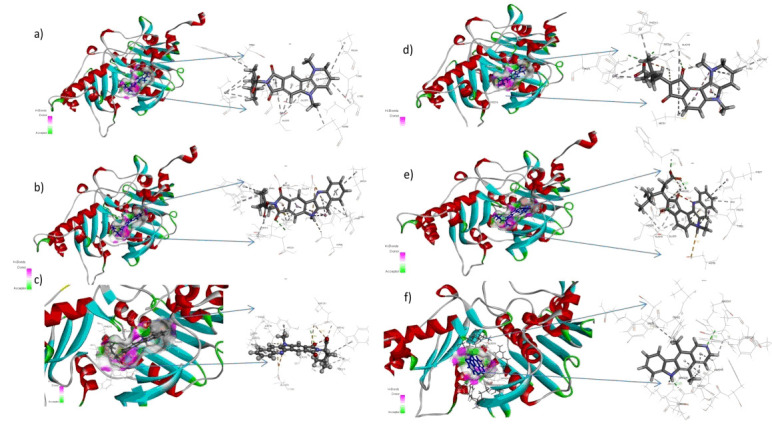
α-Topo II with libdock hit molecules.

**Figure 8:**
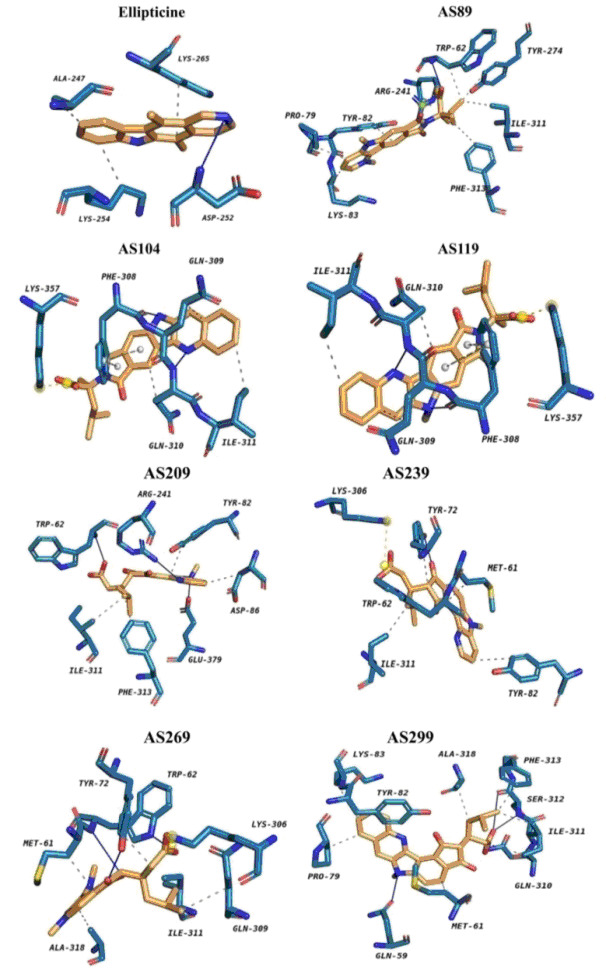
Binding interactions between ligand and receptor through grid-based docking.

Libdock high throughput docking was performed for α-Topo II for all the designed compounds (Figure 7-Figure 8). The results were compared with co-crystal ligand binding. The more positive libdock score was considered as better binding. Compounds AS89, AS104, AS119, AS209, AS239, AS269 and AS299 showed better binding activity compared to co-crystal ligand (Table 1 - see PDF). The hit molecules were docked with grid-based molecular docking (CDOCKER) and flexible docking (Autodockvina). The Cdocker energy of the seven lead compounds showed better binding potential compared to ellipticine (Table 2 - see PDF). The flexible docking results show favourable non-bond interactions, including hydrogen bond interactions and hydrophobic interactions (Table 3 - see PDF). The binding energy of the docked complex is shown in Table 2 (see PDF). Compound AS119 shows good binding energy of -9.07kj/mol compared to the known inhibitor, ellipticine (-7.91kj/mol) with three hydrophobic bonds (309GLN, 310GLN, 311ILE) and two hydrogen bonds (308PHE, 310GLN) interactions (Table 3 - see PDF & Figure 8). Salt Bridge interactions are observed in compound AS89 with 241ARG; compound AS104 and AS119 with 357LYS and compound AS239 and AS269 with 306LYS. Pi-Stacking interactions are observed in compounds AS104 and AS119 with 308PHE. All the seven complexes of α-Topo II-δ-carboline derivatives, AS89, AS104, AS119, AS209, AS239, AS269 and AS299, show better -Cdocker energy, -Cdocker interaction energy and binding energy compared to Ellipticine (Table 2 - see PDF).

### Molecular dynamic simulations:

**Figure 9:**
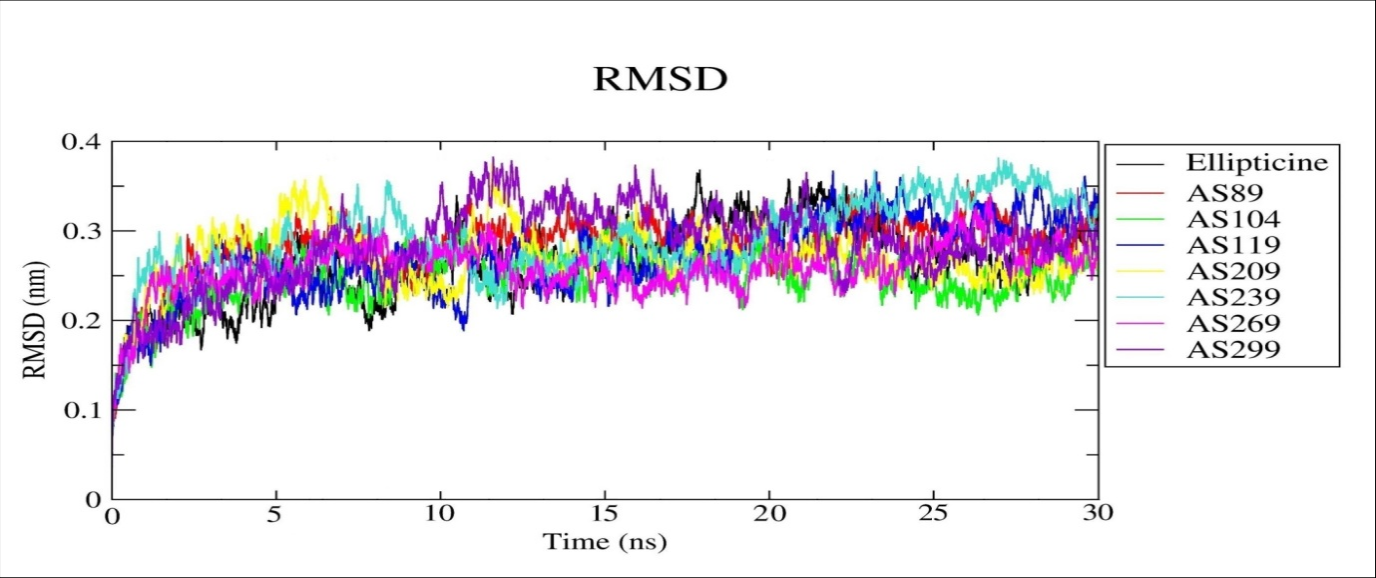
RMSD trajectories of α-Topo II-ligand complexes.

MD simulations were performed for α-Topo-II-Ellipticine, and the designed α-Topo II-ligand complexes. The 30ns MD simulation of RMSD change in the Cα atom of the protein-ligand complex is shown in the RMSD plot (Figure 9). The fluctuations of the RMSD of 0.2 to 0.4 nm suggest that the receptor and drug binding interactions stabilize in the 30ns MD simulation. The RMSD for all the compounds show different fluctuations, which increase from 0.1 nm to 0.35 nm upto 20ns and stabilize between 0.23 nm to 0.35 nm through the simulation.

**Figure 10:**
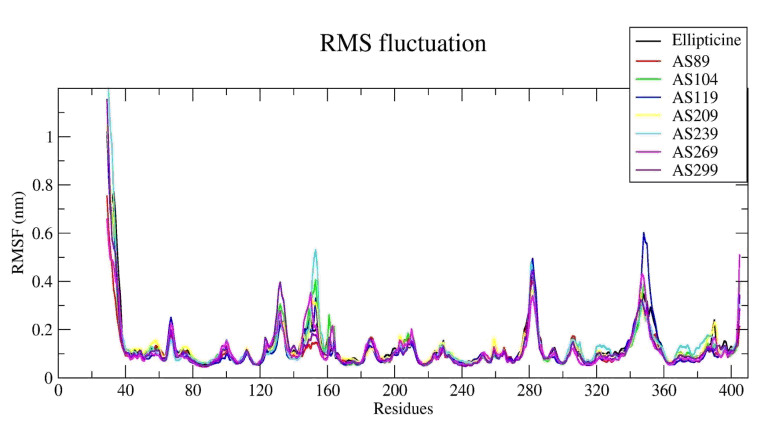
RMSF trajectories of α-Topo II –ligand complexes with fluctuation.

**Figure 11:**
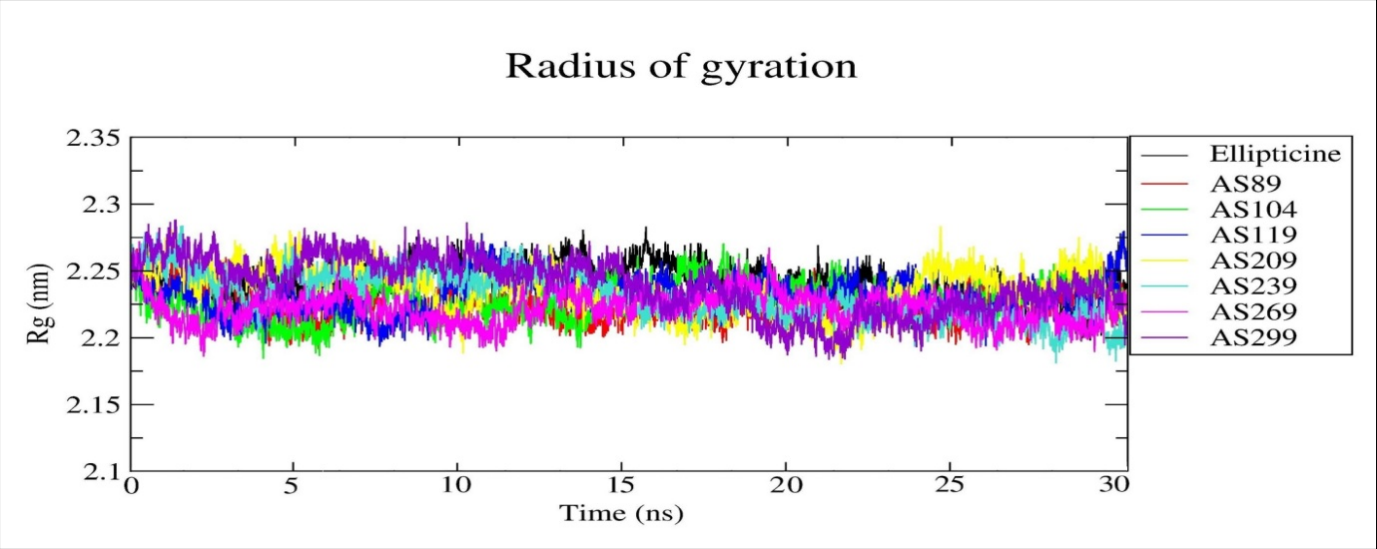
Radius of Gyration trajectories of α-Topo II-ligand complex fluctuation.

The RMSF values were plotted against residues. The plot shows more fluctuation at initial residues in the N-terminal because of the loop. Residues from120 to 170, 270 to 290 and 330 to 360 show more fluctuations. Residues between 120 to 170 show fluctuation between 0.1nm to 0.5nm and residues between 270 to 290 and 330 to 360 show more fluctuation because of the presence of hydrogen bonds. Hydrophobic interactions are responsible for the lower or higher RMSF valuesobserved in different α-Topo II-ligand complexes (Figure 10). These hydrogen bonds, hydrophobic, halogen bond, π-stacking interaction play essential roles in the fluctuation of RMSF values. Radius of gyration (Rg) analysis is used to predict the level of compactness of the protein and the ligand. The compactness was found to be good as revealed by the lower Rg values (Figure 11). The Rg value for α-topo II-ellipticine and the seven α-Topo II-ligand complexes are similar for the entire30000ps (30ns). The increase or decrease in the Rg level and hence compactness is due to the difference in the binding nature of the ligand during the simulation. The Rg plot reveals that compounds bind to protein with good compactness.

### Binding free energy analyses:

Binding free energy calculation from simulation approaches is a most accurate strategy to substantiate the binding of compounds with favourable thermodynamics. The binding free energies of all the compounds were calculated by MM/PBSA methods. All compounds show negative binding energy (Table 4 - see PDF) Among the seven lead compounds and Ellipticine for which binding free energies were calculated, compound AS269 shows a positive binding energy of 22.641kJ/mol. AS104 is seen to be the most favourable due to lower Van der Waals (-124.857kJ/mol) and electrostatic (-37.213 kJ/mol) interaction energies that provide better binding. Furthermore, electrostatic, nonpolar solvation energy, and Van der Waals interactions negatively compliment the overall interaction energy. Polar and nonpolar negative free energy also play an essential role for binding of the protein. Compound AS299 has a higher SASA (solvent accessible surface area) (-19.030kJ/mol) among all the compounds. Calculated free energies suggest that the Van der Waal energy and Electrostatic energy play a crucial role in the ligands binding to α-Topo II (Table 4 - see PDF).

## Conclusion:

The α-Topo II inhibitors play a crucial role in curing breast cancer, ovarian cancer, and colon cancer. Three hundred δ-carboline derivatives fused with pyrrolidine-2,5-dione were designed and analysed using molecular docking techniques with a 30 ns simulation for the evaluation of suitable features such as drug-likeness properties as well as ADMET properties. The binding free energy calculations selected seven of these molecules is novel inhibitors of α-Topo II for further consideration.
